# Effect of Nano-Silver Fluoride Containing 2000 ppm Silver Nanoparticles on the Microhardness and Color of Flowable Resin Composites: An In Vitro Pilot Study

**DOI:** 10.3390/ma19153291

**Published:** 2026-08-03

**Authors:** Emanuel Ewerton Mendonça Vasconcelos, Maria Clara Müller de Andrade, André Galembeck, Gabriela Queiroz de Melo Monteiro, Fernanda Donida Ortigoza, Luís Felipe Espíndola-Castro

**Affiliations:** 1 Faculty of Dentistry, Federal University of Pernambuco (UFPE), Recife 50670-901, Brazil; emanuel.ewerton@ufpe.br (E.E.M.V.); fernanda.adonida@ufpe.br (F.D.O.); 2 Department of Fundamental Chemistry, Federal University of Pernambuco (UFPE), Recife 50670-901, Brazil; clara.muller@ufpe.br (M.C.M.d.A.); andre.galembeck@ufpe.br (A.G.); 3 Faculty of Dentistry, University of Pernambuco (UPE), Recife 50040-200, Brazil; gabriela.queiroz@upe.br

**Keywords:** flowable resin composites, nano-silver fluoride, silver nanoparticles, antimicrobial agent

## Abstract

Although the modification of resin composites with antimicrobial agents represents a viable alternative for biofilm inhibition, these changes may compromise the optical and mechanical properties of dental restorations. This in vitro pilot study evaluated the effect of incorporating Nano-Silver Fluoride (NSF) containing 2000 ppm silver nanoparticles (AgNPs) on the microhardness and color of flowable resin composites: Vittra APS Unique Flowable (VA), Filtek Bulk Fill Flowable (FB), and Filtek Supreme Flowable (FS). The resin composites were modified by adding 1 wt%, 2 wt%, and 4 wt% NSF and compared with an unmodified group (control). NSF was characterized by UV–visible spectroscopy and transmission electron microscopy. The specimens were subjected to Vickers microhardness testing and color analysis using the CIEDE2000 (ΔE_00_) and CIELAB (ΔE) systems. NSF incorporation had a material-dependent effect on microhardness. The VA group showed a progressive reduction in microhardness with increasing NSF additions, whereas the FS group remained unchanged up to the concentration of 2 wt%, and the FB group showed no significant reduction in microhardness at any of the tested concentrations compared with the control (*p* = 0.549). All resin composites exhibited clinically relevant color changes that increased with the NSF concentration, with more pronounced color changes in the VA group, whereas FB and FS did not vary significantly with the addition of different NSF concentrations. Overall, NSF mainly affected the optical properties, with esthetic limitations being the main challenge to be overcome in further studies. The FB and FS groups at 1 wt% and 2 wt% showed smaller chromatic changes than VA, without compromising microhardness, and appear promising for future investigations, including antimicrobial and cytotoxicity analyses.

## 1. Introduction

Resin composites are first-choice materials for dental restorations and have been widely used as an alternative to silver amalgam [1]. Resin composites exhibit superior esthetics, easy handling, adhesion to tooth structures, and greater biocompatibility compared with silver amalgam restorations [2,3]. However, the popularity of this material faces a critical challenge: polymerization shrinkage, hydrolytic degradation, and differences in the coefficient of thermal expansion between the resin composite and the tooth may lead to gap formation at the tooth–restoration interface [4]. These spaces can be colonized by oral bacteria and are associated with the occurrence of microleakage and the development of secondary caries, one of the main factors related to failures in resin composite restorations [1,5,6].

In this context, various strategies have been investigated to minimize secondary caries rates, including modifying restorative materials to confer antimicrobial properties [7], incorporating bioactive agents [8], and using nanomaterials with antibacterial activity [9]. Silver nanoparticles (AgNPs), for example, exhibit a high surface area-to-volume ratio [10], which is associated with significant antimicrobial activity [11,12], even at low concentrations [13].

Nano-Silver Fluoride (NSF) is a compound comprising AgNPs, fluoride, and chitosan, and has been investigated as a strategy for caries prevention and control [14]. Its effect is based on the combination of fluoride’s remineralizing potential and the antimicrobial properties of silver nanoparticles [12]. AgNPs can exert antibacterial activity through the release of silver ions (Ag^+^), the generation of reactive oxygen species (ROS), and alterations in the bacterial cell membrane [13]. Chitosan, in turn, is a cationic, biocompatible, and biodegradable biopolymer [15] whose molecular structure enables it to interact with negatively charged bacterial membranes [16]. Chitosan may exhibit synergistic effects that contribute to the antimicrobial and remineralizing performance of NSF [17].

In resin-based materials, incorporating nanoparticles can modify mechanical and optical properties, and these effects depend on particle type, concentration, and dispersion within the matrix [18]. The effects of AgNPs incorporation on mechanical properties remain inconsistent, with some studies reporting improvements [19,20,21] and others showing no reductions in certain properties [22,23,24]. The incorporation of AgNPs into resin composites may reduce microleakage and influence mechanical properties, such as microhardness, depending on the concentration used [24].

Despite the potentially beneficial effects reported, the addition of bioactive components to resin composites may also have undesirable effects, including color alteration, which may compromise the esthetic outcome of restorations. Some studies have evaluated the incorporation of selenium nanoparticles (SeNPs) [25], graphene oxide nanoparticles (GONPs) [26], titanium dioxide nanoparticles (TiO_2_NPs), zirconium dioxide nanoparticles (ZrO_2_ NPs), silicon dioxide nanoparticles (SiO_2_ NPs) [27], and silica-coated silver nanoparticles (Ag@SiO_2_ NPs) [28]. However, no study has evaluated the incorporation of NSF into resin composites.

Therefore, the aim of this in vitro pilot study was to evaluate the influence on the microhardness and color stability of three flowable resin composites: Vittra APS Unique Flowable (FGM, Joinville, Brazil), Filtek Bulk Fill Flowable Restorative (Solventum, St. Paul, MN, USA), and Filtek Supreme Flowable Restorative (Solventum, MN, USA), after the incorporation of NSF containing 2000 ppm AgNPs at concentrations of 0% (control), 1 wt%, 2 wt%, and 4 wt%. This preliminary study was designed to identify the most promising concentrations of the tested product to enable future analyses of the antimicrobial potential and cytotoxicity of the proposed modification. The null hypotheses tested were as follows: (I) the addition of NSF does not influence the microhardness of the tested resin composites; (II) the addition of NSF does not influence the color of the resin composites; (III) different NSF concentrations do not influence the microhardness or color of the tested resin composites; and (IV) the tested resin composites behave similarly after NSF incorporation.

## 2. Materials and Methods

This in vitro pilot study evaluated three flowable resin composites: Vittra APS Unique Flowable (FGM Dental Group, Joinville, SC, Brazil), Filtek Bulk Fill Flowable Restorative (Solventum, St. Paul, MN, USA), and Filtek Supreme Flowable Restorative (Solventum, St. Paul, MN, USA). For the experimental design, the resin composites were modified by incorporating a Nano-Silver Fluoride solution at a concentration of 2000 ppm. The detailed chemical composition, manufacturers’ specifications, and batch numbers of all materials used in this study are summarized in Table 1.

### 2.1. Sample Size

For the sample size calculation, a previous study that evaluated microhardness and color changes in resin composites was considered. To achieve statistically significant differences in microhardness between the groups, a difference of 4.4 HV was required [29]. Based on a mean standard deviation of ±2.22 from the study, a significance level of 5%, and a power of 80%; the minimum sample size per group was 6. Considering possible losses during the analysis, the number of samples was increased by 30%, and *n* = 8 per group was adopted. For the sample size calculation, the test for comparing more than two means from independent groups (ANOVA) was considered, using the sample size calculator available on the University of São Paulo website: http://estatistica.bauru.usp.br/calculoamostral/calculos.php (accessed on 10 January 2026).

### 2.2. Synthesis and Characterization of NSF

The synthesis of the NSF used in this study followed a route previously described in the literature [30]. In the first step, a chitosan solution (0.01 g·mL^−1^) was prepared in 1% (*v*/*v*) acetic acid, to which an aqueous silver nitrate solution (AgNO_3_; 0.11 mol·L^−1^) was added. Subsequently, the reducing agent sodium borohydride (NaBH_4_; 0.66 mol·L^−1^) was added to form silver nanoparticles (AgNPs), resulting in yellow colloidal suspension. Sodium fluoride (NaF; 8.39 mol·L^−1^) was then incorporated into the system under stirring until the salt was completely dissolved.

The optical characterization of NSF was performed using UV–visible absorption spectroscopy with a Cary 60 UV–Vis spectrophotometer (Agilent, Santa Clara, CA, USA) and 1 cm quartz cuvettes. The scan was conducted over the range of 300 to 1000 nm, using deionized water as the reference standard. The AgNPs were analyzed by transmission electron microscopy (TEM) using a Talos F200i microscope (ThermoScientific, Waltham, MA, USA) operating at 200 kV. For this step, one drop of NSF diluted in deionized water was deposited onto a carbon-coated copper grid and allowed to dry at room temperature. The obtained images were processed using ImageJ software (version 1.54g; NIH, Bethesda, MD, USA) to automatically measure particle size (n > 1200).

### 2.3. NSF Incorporation and Specimen Preparation

For each commercial resin composite brand (Table 1), three distinct experimental groups were established, varying according to the proportion of NSF added: 1 wt%, 2 wt%, and 4 wt%, compared with the control group (resin composite without NSF addition). The selected concentrations were chosen to provide a preliminary dose–response evaluation across a broad range of NSF incorporation in this pilot study, allowing the identification of concentration-dependent effects on the physicochemical properties of the resin composites. Considering that the aqueous NSF solution has a density of approximately 1.0 g/mL, incorporation was performed by pipetting 5 µL, 10 µL, and 20 µL of the NSF solution, respectively, for each 0.5 g (500 mg) of resin composite.

**Figure 1:**
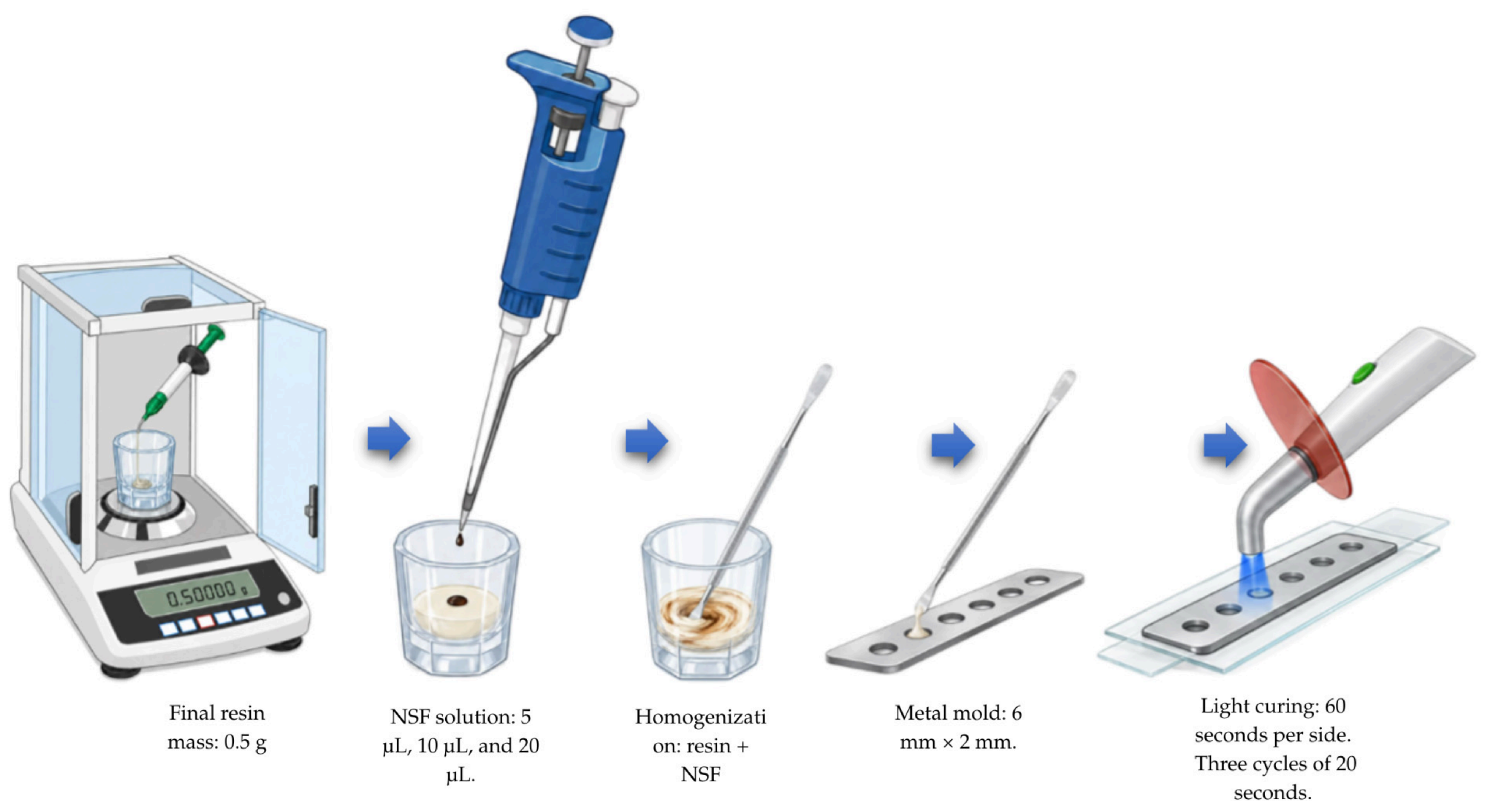
Specimen preparation and curing protocol. Specimen preparation and curing protocol (created using ChatGPT, OpenAI GPT-5.5).

The process of incorporating NSF into the resin matrix is schematized in Figure 1. To ensure sample standardization, the resin composite was weighed on an analytical balance with a precision of +0.01 mg (AUW 220D, Shimadzu, Kyoto, Japan). A previously tared glass container was used, to which the resin was added until the final mass reached 0.5 g. Then, using a micropipette, 5 µL, 10 µL, and 20 µL of the NSF solution was dispensed into 0.5 g of resin composite, corresponding to 1 wt%, 2 wt%, and 4 wt%, respectively. The mixture was manually spatulated (Spatula No. 7, Golgran, São Caetano do Sul, SP, Brazil) until complete homogenization, resulting in a visually homogeneous mass free of bubbles and streaks.

After homogenization, the modified resin composite was inserted into a metal mold (6 mm in diameter and 2 mm in thickness). Polyester strips and glass slides were positioned on both sides of the mold under constant pressure (500 g glass plate) to extrude excess material, flatten the surface, and ensure the exact thickness of the specimens.

Light curing was performed using a Radii-Cal light-curing unit (SDI Limited, Bayswater, VIC, Australia), operating at an irradiance of 1200 mW/cm^2^ and a wavelength between 440 and 480 nm (peak at ~460 nm). The device’s irradiance was previously measured using a radiometer (SDI Radiometer X, Bayswater, VIC, Australia). Photoactivation was performed in direct contact with the glass slide for 60 s on each side of the disc, divided into three consecutive 20 s cycles. After preparation, the specimens were removed from the mold, inspected for structural defects, and stored in Falcon-type tubes (Kasvi, São José dos Pinhais, PR, Brazil).

### 2.4. Vickers Microhardness Evaluation (VHN)

The Vickers microhardness test (VHN) was performed using a digital microhardness tester (ISH-MR 150/INSIZE, São Paulo, Brazil). For each specimen (*n* = 8), the indenter was applied to the central region under a load of 300 gf for 15 s. Three indentations were made on each sample, spaced 3 mm apart, and the arithmetic mean of these three readings was adopted as the representative microhardness value of the sample.

### 2.5. Color Evaluation

The color evaluation of the specimens after NSF incorporation was performed by a single previously calibrated examiner using the cross-polarized (CP) photographic method, adapted from protocols described in the literature [31,32].

The images were captured using a DSLR digital camera (EOS Rebel T7+, Canon, Tokyo, Japan), equipped with a macro lens (100 mm, f/2.8, Canon, Tokyo, Japan) and a twin-flash lighting system (YN24EX, Yongnuo, Shenzhen, China). To obtain the cross-polarization effect and eliminate specular reflection, linear polarizers were adapted to the flashlight outputs together with a circular polarizing filter (K&F Concept, Shenzhen, China) attached to the objective lens.

To ensure reproducibility of captures, the camera parameters were set to manual mode: ISO 200, aperture f/16, shutter speed 1/200 s, and white balance was set to “Custom”. The photographic environment was standardized using a black fabric photographic tent (light box) to completely block interference from external lighting. The specimens were positioned on a standardized background (18% gray card) and photographed at a fixed distance of 30 cm from the objective lens. To ensure stability and precise maintenance of this working distance across all captures, the camera was mounted on an aluminum tripod (WT 3750, Greika, São Paulo, SP, Brazil) and used manual focus only.

The standardized photographic images were opened individually in Adobe Photoshop CC 2019 software (Adobe Systems, San Jose, CA, USA) for quantitative extraction of the color coordinates. Using the Color Sampler Tool together with the Info panel, the central region of interest of each specimen was selected for reading the chromatic parameters. The mean values were recorded based on the CIELab color space, whose axes express: L* (lightness, ranging from 0 = black to 100 = white), a* (green–red chromatic axis), and b* (blue–yellow chromatic axis).

The overall color changes ΔE_00_ and ΔE caused by NSF incorporation between the experimental groups and the control group (without NSF addition) were calculated using the CIEDE2000 and CIELab formulas:

CIEDE2000:

$$\Delta E_{00}=\sqrt{{\left(\frac{\Delta L^{\prime }}{K_{L}S_{L}}\right)}^{2}+{\left(\frac{\Delta C^{\prime }}{K_{C}S_{C}}\right)}^{2}+{\left(\frac{\Delta H^{\prime }}{K_{H}S_{H}}\right)}^{2}+R_{T}\left(\frac{\Delta C^{\prime }}{K_{C}S_{C}}\right)\left(\frac{\Delta H^{\prime }}{K_{H}S_{H}}\right)}$$

where

ΔL′ = difference in lightness between the samples;
ΔC′ = difference in chroma between the samples;
ΔH′ = difference in hue between the samples;
S_L_ = lightness weighting function;
S_C_ = chroma weighting function;
S_H_ = hue weighting function;
K_L_ = parametric correction factor for lightness;
K_C_ = parametric correction factor for chroma;
K_H_ = parametric correction factor for hue;
R_T_ = rotation term representing the interaction between chroma and hue differences in the blue region of the CIELAB color space.

CIELab:

$$\Delta E=\sqrt{{\left(L_{2}^{*}-L_{1}^{*}\right)}^{2}+{\left(a_{2}^{*}-a_{1}^{*}\right)}^{2}+{\left(b_{2}^{*}-b_{1}^{*}\right)}^{2}}$$

where

ΔL* = difference in lightness between the samples;
Δa* = difference along the green–red axis;
Δb* = difference along the blue–yellow axis.

To interpret the clinical relevance of the optical changes, the color difference values were analyzed using the perceptibility and acceptability thresholds established in the literature [33]. For the CIEDE2000 formula, values of ΔE_00_ = 0.81 for clinical perceptibility (PT) and ΔE_00_ = 1.80 for clinical acceptability (AT) were considered. For the CIELAB system, thresholds of ΔE_ab_ = 1.2 for clinical perceptibility (PT) and ΔE_ab_ = 2.7 for clinical acceptability (AT) were adopted.

### 2.6. Statistical Analysis

The data were presented using measures of central tendency and dispersion, namely mean and standard deviation. The normality of the distribution was verified using the Shapiro–Wilk test, and the homogeneity of variances using Levene’s test.

For comparisons between resins at each concentration or between concentrations within each resin, the F test (ANOVA) or the Kruskal–Wallis test was applied. When significant differences were identified, multiple comparisons were performed using Tukey’s test when the test indicated equality of variances, or Tamhane’s test when equality of variances was rejected. If a significant difference was identified by the Kruskal–Wallis test, multiple comparisons were performed using Conover’s test.

The one-way ANOVA was selected in situations in which the data showed a normal distribution, whereas the Kruskal–Wallis test was used when the hypothesis of normality was rejected in at least one of the categories.

The significance level adopted was 5%. The analyses were performed using IBM SPSS version 27 and MEDCALC version 20.104 software.

## 3. Results

### 3.1. Characterization of NSF

**Figure 2:**
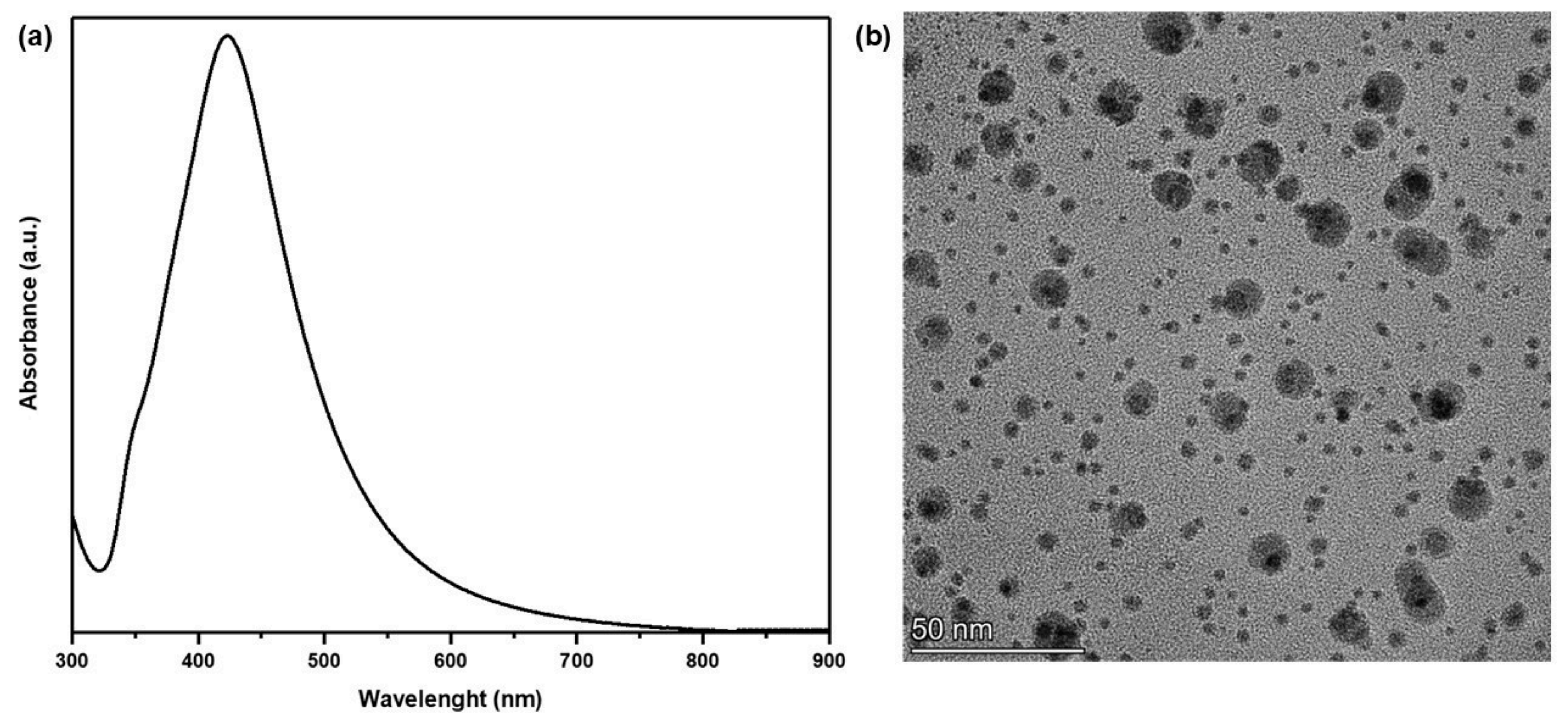
Characterization of nano-silver fluoride (NSF). (**a**) UV–visible absorption spectrum of NSF. (**b**) Transmission electron microscopy (TEM) image of NSF showing the morphology and distribution of the silver nanoparticles.

Figure 2 shows the absorption spectrum in the UV-visible region of the NSF used in this study, overlaid with a TEM image of the silver nanoparticles that compose the formulation. The synthesized nanoparticles exhibited a predominantly spherical morphology, with a mean diameter of 5.24 ± 4.12 nm. The UV–Vis spectrum exhibited a localized surface plasmon resonance (LSPR) band with λmax at 425.9 nm and a full width at half maximum (FWHM) of 126.5 nm.

The position of this band is consistent with the presence of silver nanoparticles, as previously reported in the literature [34,35,36]. In this context, the slight red shift observed (425.9 nm) may be related both to the presence of relatively larger particles in the distribution and to possible plasmonic coupling effects resulting from partial aggregation, corroborating the findings observed in the microscopic analysis.

### 3.2. Vickers Microhardness (VHN)

Table 2 presents the microhardness results for the VA, FB, and FS resin composites at the tested concentrations. A significant effect of NSF concentration on the microhardness of the VA resin composite was observed (*p* < 0.001). The highest microhardness value for the VA resin composite was recorded in the Control group (43.94 ± 1.89). As NSF concentration increased, microhardness progressively decreased, with significant differences among all groups compared with the control. For the FB resin composite, no significant effect of NSF concentration on microhardness was observed. Microhardness values ranged from 32.93 ± 1.49 in the Control group to 31.52 ± 2.28 at 4 wt%, with no significant differences among concentrations (*p* = 0.549).

For the FS resin composite, NSF concentration also had a significant effect on microhardness (*p* < 0.001). The highest means were observed at 1 wt% and 2 wt%, with values of 43.99 ± 1.53 and 42.64 ± 2.13, respectively. The Control group showed a mean of 40.88 ± 2.71, whereas the lowest value was recorded at the concentration of 4 wt% (38.42 ± 1.64). According to Tukey’s test, only the 4 wt% concentration differed significantly from the other groups. When the resin composites were compared within each concentration, significant differences were observed (*p* < 0.001).

### 3.3. Color Evaluation

Table 3 presents the color difference (∆E_00_) and (∆E) values according to resin composite and NSF concentration. For the ΔE_00_ analysis, significant differences among the resin composites were observed at all tested concentrations (*p* < 0.001). At 1 wt%, the VA resin composite showed a mean ∆E_00_ value of 12.51, whereas the FB and FS resins had means of 8.92 and 8.91, respectively. At 2 wt%, the mean values were 14.42 for VA, 8.96 for FB, and 9.86 for FS. At 4 wt%, the corresponding values were 13.43, 8.52, and 9.06, respectively. Regarding the effect of NSF concentration within each resin composite, significant differences were observed only for the VA resin composite (*p* = 0.011), with the highest ΔE_00_ value observed at 2 wt%. No significant differences were found among the tested concentrations for the FB and FS resin composites.

A similar pattern was observed for the CIELAB (ΔE) analysis. However, higher color difference values were observed for all resin composites and NSF concentrations, as shown in Table 4.

The color changes are illustrated by the representative photographic records presented in Table 4.

## 4. Discussion

The incorporation of NSF into the evaluated resin composites significantly influenced the optical properties and, in a material-dependent manner, the microhardness of the tested resin composites. The first null hypothesis, that the addition of NSF would not influence resin composite microhardness, was rejected. For the VA resin composite, microhardness decreased progressively with increasing NSF concentration. In the FS resin composite, microhardness remained high at concentrations of 1 wt% and 2 wt%, with a significant reduction only at 4 wt%. In contrast, the FB resin composite showed no significant differences across groups. These findings may be related to the formulation of each material. Studies have shown that microhardness depends mainly on filler composition and content, especially by weight [37], and reinforce that larger particles in resin composites tend to result in higher surface hardness [38]. The VA resin composite has the highest percentage of organic matrix, and the addition of NSF further reduces the percentage of filler particles. These findings may help explain the stability of FB microhardness and the maintenance of higher microhardness values in FS, since both contain 65% filler particles by weight. Additionally, previous findings have shown that the type of base monomer influences this property, with UDMA associated with greater hardness than Bis-GMA or Bis-EMA [39].

Moreover, in the VA resin composite, the reduction in microhardness may be associated with a greater sensitivity of its light-curing system to changes induced by NSF. Resin composites with greater dependence on the organic matrix tend to be more susceptible to structural modifications resulting from the incorporation of additional particles [40,41]. It is also worth noting that VA contains the APS system, characterized by a lower concentration of camphorquinone and secondary photoinitiators, which may favor monomer conversion but also make the material more dependent on adequate light transmission [42].

Based on this mechanism, NSF incorporation may have reduced light transmission at wavelengths relevant to photoactivation, potentially leading to a lower degree of conversion and, consequently, reduced crosslink density and microhardness [28,43]. This interpretation is consistent with previous studies showing that silver-containing particles can reduce the degree of conversion and compromise mechanical properties, particularly at higher concentrations or when light propagation during photoactivation is impaired [44,45]. Future studies should assess the degree of conversion by FTIR to determine whether NSF incorporation affects polymerization efficiency and to explain the microhardness differences observed in the present study.

In contrast, the FB and FS resin composites may have exhibited greater stability during the incorporation of silver nanoparticles due to their composition, filler content, and conventional camphorquinone-based photoinitiator system. The effectiveness of light curing depends on the interaction among the photoinitiator, the light source’s emission spectrum, and the light transmission within the material [46]. Although silver particles may interfere with light propagation, the intrinsic characteristics of the FB and FS resin composites may have favored maintenance of microhardness.

Regarding color, the second null hypothesis, that the addition of NSF would not influence the resin composites, was also rejected, since significant differences were observed for the ΔE_00_ and ΔE variables. In both evaluation systems, the VA resin composite exhibited the highest mean color change across all tested concentrations. The FB and FS resin composites showed lower color difference values and greater stability across the tested concentrations. Only the VA group showed a significant difference among concentrations, with an increase in color change at 2 wt%, but no significant difference compared with 4 wt%. This finding also led to the rejection of the third null hypothesis, since significant differences were observed when different NSF concentrations were applied.

The interpretation of these values should consider the chromatic perceptibility and acceptability thresholds described for dental materials. Paravina et al. (2015) observed that, for the CIEDE2000 system, the perceptibility threshold is ΔE_00_ = 0.8 and the acceptability threshold is ΔE_00_ = 1.8; whereas, for the CIELAB system, the corresponding values are ΔE_ab_ = 1.2 and ΔE_ab_ = 2.7, respectively [33]. These thresholds enable distinguishing changes that are only visually detectable from those considered clinically unacceptable, serving as parameters for the selection, evaluation, and quality control of esthetic dental materials. In the present study, the ΔE_00_ and ΔE values observed for all evaluated resin composites and concentrations exceeded the specified acceptability limits, indicating that NSF incorporation promoted clinically relevant chromatic alteration, particularly in the VA resin composite, which exhibited the highest values in both evaluation systems.

However, it is important to emphasize that the perceptibility and acceptability values reported in the literature generally refer to color changes that a material undergoes over time. In the present study, however, the color change resulting from the incorporation of NSF into the resin composite was evaluated before the restorative procedure. Thus, the final color of the material is already established before clinical use. As shown in Table 4, the initial color of the resin composites, according to the Vita Classical and Vita 3D-Master shade guides, is presented, along with the color observed after incorporating different NSF concentrations. Therefore, the color changes observed after NSF incorporation could potentially be compensated for during clinical shade selection. For example, adding 2 wt% NSF to the FS and FB resin composites changed the shade from A3 to A4.

In addition, the present study evaluated the immediate effects of NSF incorporation without artificial aging. In the oral environment, thermal cycling, water sorption, and pH fluctuations may accelerate the release of silver ions and other NSF components from the resin matrix. Although this sustained release may prolong the antimicrobial effect, it could also influence the material’s long-term stability by altering its optical and mechanical properties. Therefore, future studies should investigate the behavior of NSF-modified resin composites after thermomechanical aging and prolonged water storage to determine whether the immediate findings observed in the present study are maintained over time.

The greater color changes in the VA resin composite may be mainly related to its less saturated shade (OM2/B1), which may be more easily modified by the addition of NSF. Furthermore, Tapety et al. (2023) highlighted that this material has a distinct photoinitiator system, in addition to its own matrix and filler particle composition [42]. Recent studies have shown that the type, size, and amount of inorganic particles, as well as the refractive-index mismatch between the organic matrix and fillers, affect light transmission and the optical properties of resin composites [41]. This suggests that the presence of NSF may have altered light transmission, scattering, and absorption in VA, resulting in a greater color impact.

This behavior may also be explained by the metallic nature of silver nanoparticles, which have a high capacity to absorb and scatter light, modifying the translucency and luminosity of the material [47]. Previous studies have shown that incorporating silver nanoparticles into a resin matrix reduced transmittance and induced perceptible color change [28]. Similarly, another study reported color changes in resin composites containing silver-releasing filler particles [45]. Therefore, differences in translucency, organic matrix, APS system, pigment content, and the optical relationship between matrix and particles may have reduced VA’s masking capacity against the change promoted by NSF. These results were less pronounced in the FB and FS resin composites; therefore, the fourth null hypothesis was also rejected, since significant differences were observed among the tested resin composites.

The absence of significant differences among the 1 wt%, 2 wt%, and 4 wt% concentrations in the FB and FS resin composites suggests that low NSF concentrations were sufficient to induce optical alteration in these materials. At higher concentrations, possible nanoparticle agglomeration may have limited their homogeneous dispersion in the resin matrix, reducing the proportional increase in color alteration [48]. This behavior reinforces that the optical effects of metallic nanoparticles depend not only on the amount incorporated but also on their distribution, interaction with the matrix, and ability to modify light transmission and scattering.

From a clinical perspective, the relevance of the reduction in microhardness and the color alteration should be interpreted according to the indication of flowable resin composites. Although highly filled flowable resin composite formulations currently exist and are indicated even for definitive restorations, these materials continue to be widely used as bases, cavity liners, for regularization of internal walls, and as intermediate layers under higher-viscosity resin composites, due to their high flowability and adaptation to the substrate [49]. Therefore, the changes in color and microhardness observed in the present study could be compensated for by covering this material with conventional resin composites, while maintaining the antimicrobial properties that NSF may provide to resin composites.

Clinical studies have demonstrated that restorations using flowable resin composites as a base covered by conventional resin composites show satisfactory performance similar to that of conventional restorative techniques [50,51].

The findings of this study also indicate that the NSF concentration used should be carefully considered. Studies involving metallic nanoparticles in resin composites have demonstrated concentration-dependent behavior, in which low concentrations may preserve or even improve certain properties, whereas higher concentrations may favor particle agglomeration, increased opacity, reduced light transmission, and impairment of mechanical properties [28,44,45]. Thus, NSF concentrations below 2000 ppm AgNPs may represent a promising strategy for future studies, as they reduce optical changes while preserving the material’s mechanical and antimicrobial properties.

However, this hypothesis still requires confirmation through additional studies evaluating lower AgNPs concentrations, degree of polymer conversion, sorption, solubility, as well as confirming the antimicrobial and biocompatibility properties of the proposed resin composite modifications. Thus, although NSF shows potential for modifying flowable resin composites, its incorporation should be adjusted to balance antimicrobial activity, optical stability, and maintenance of mechanical properties.

Within the limitations of the present study, its in vitro nature should be mentioned, as it does not fully reproduce the complexity of the humid environment, thermal challenges, pH variations, and biomechanical stresses of the oral cavity. In addition, the mechanical and optical properties were evaluated only immediately, without subjecting the samples to artificial aging processes, such as thermal and mechanical cycling, as well as hydrolytic degradation, which could alter the behavior of the material over time. Future studies should investigate the degree of conversion (FTIR), water sorption and solubility, UV–Vis light transmission, contact angle measurements, and microscopic characterization of nanoparticle dispersion (SEM/EDS or TEM) to provide a more comprehensive understanding of the compatibility between NSF and the resin matrix.

## 5. Conclusions

Based on the results of this in vitro pilot study, incorporating NSF containing 2000 ppm AgNPs influenced the color and, in a material-dependent manner, the microhardness of the evaluated resin composites. These findings suggest that incorporating low NSF concentrations into FB and FS may represent a promising strategy for future antimicrobial investigations, while preserving microhardness and reducing color changes compared with VA. Furthermore, lower AgNP concentrations incorporated into the resin composite may represent a promising strategy to minimize the esthetic limitations observed in this study and should be investigated in future studies.

## Figures and Tables

**Table 1:** Groups, resin composites tested and composition.

Group	Materials	Shade	Composition	Classification	Batch
VA	Vittra APS Unique Flow (FGM Dental Group, Joinville, SC, Brazil)	Monochromatic	Silanized barium-aluminum-borosilicate glass and silanized silica dispersed in a methacrylate monomer matrix. APS (Advanced Polymerization System) photoinitiator system with a low concentration of camphorquinone. Filler particle content: approximately 58–62 wt% and 56–60 vol%. BPA-free material.	Submicrometric flowable resin composite.	140525
FB	Filtek Bulk Fill Flowable Restorative (Solventum, St. Paul, MN, USA)	A2	Treated silanized ceramic, treated silica, Bis-GMA, Bis-EMA(6), UDMA, PEGDMA, silane-treated zirconia, and TEGDMA. Filler particle content: 65 wt% and 42.4–42.5 vol%.	Nanoparticle-filled flowable resin composite.	2510700685
FS	Filtek Supreme Flowable Restorative (Solventum, St. Paul, MN, USA)	A2	Treated silanized ceramic, substitute dimethacrylate, Bis-GMA, treated silica, TEGDMA, Poly[oxy(1-oxo-1,6-hexanediyl)], α,α′-(oxydi-2,1-ethanediyl)bis[ω-[[[[2-[(2-methyl-1-oxo-2-propen-1-yl)oxy]ethyl]amino]carbonyl]oxy]-, ytterbium fluoride, N, N-dimethylbenzocaine, and diphenyliodonium hexafluorophosphate. Filler particle content: 65 wt% and 46 vol%.	Nanoparticle-filled flowable resin composite.	2513400543
NSF	Nano-Silver Fluoride (2000 ppm)		Silver nanoparticles, fluoride, chitosan, water.	-	-

UDMA: Urethane dimethacrylate; Bis-GMA: Bisphenol A glycidyl methacrylate; Bis-EMA-6: Ethoxylated bisphenol A dimethacrylate; TEGDMA: Triethylene glycol dimethacrylate; PEGDMA: Polyethylene glycol dimethacrylate.

**Table 2:** Vickers microhardness (HV) according to the resin and concentration used.

Group	Concentration	Mean ± SD
VA	Control	43.94 ± 1.89 ^(A, a)^
	1 wt%	39.59 ± 1.02 ^(B, a)^
	2 wt%	34.43 ± 1.13 ^(C, a)^
	4 wt%	33.89 ± 1.98 ^(C, a)^
*p*-value		*p* ^(1)^ < 0.001 *
FB	Control	32.93 ± 1.49 ^(A, b)^
	1 wt%	32.68 ± 2.32 ^(A, b)^
	2 wt%	32.51 ± 2.01 ^(A, a)^
	4 wt%	31.52 ± 2.28 ^(A, a)^
*p*-value		*p* ^(1)^ = 0.549
FS	Control	40.88 ± 2.71 ^(A, c)^
	1 wt%	43.99 ± 1.53 ^(A, c)^
	2 wt%	42.64 ± 2.13 ^(A, b)^
	4 wt%	38.42 ± 1.64 ^(B, b)^
*p*-value		*p* ^(1)^ < 0.001 *

^(1)^ One-way ANOVA with Tukey’s post hoc test. Different uppercase letters indicate significant differences among NSF concentrations within the same resin composite, and different lowercase letters indicate significant differences among resin composites within the same NSF concentration. * Statistically significant.

**Table 3:** CIEDE2000 (ΔE_00_) and CIELAB (ΔE) values according to resin composite and NSF concentration (2000 ppm).

Evaluation	Concentration	Resin Composites			
		VA Mean ± SD	FB Mean ± SD	FS Mean ± SD	*p*-Value
ΔE_00_	1 wt%	12.51 ± 1.21 ^(A, a)^	8.92 ± 0.72 ^(B)^	8.91 ± 1.21 ^(B)^	*p* ^(1)^ < 0.001 *
	2 wt%	14.42 ± 1.28 ^(A, b)^	8.96 ± 0.41^(B)^	9.86 ± 1.02 ^(C)^	*p* ^(1)^ < 0.001 *
	4 wt%	13.43 ± 0.90 ^(A, ab)^	8.52 ± 0.53 ^(B)^	9.06 ± 1.72 ^(B)^	*p* ^(1)^ < 0.001 *
*p*-value		*p* ^(1)^ = 0.011 *	*p* ^(2)^ = 0.207	*p* ^(1)^ = 0.338	
ΔE	1 wt%	24.18 ± 2.43 ^(A, a)^	19.61 ± 2.19 ^(B)^	15.94 ± 2.28 ^(C)^	*p* ^(1)^ < 0.001 *
	2 wt%	27.69 ± 1.68 ^(A, b)^	19.34 ± 1.01 ^(B)^	17.72 ± 1.49 ^(B)^	*p* ^(1)^ < 0.001 *
	4 wt%	25.35 ± 1.42 ^(A, ab)^	18.04 ± 2.03 ^(B)^	15.43 ± 2.70 ^(B)^	*p* ^(1)^ < 0.001 *
*p*-value		*p* ^(1)^ = 0.004 *	*p* ^(1)^ = 0.209	*p* ^(1)^ = 0.119	

^(1)^ One-way ANOVA with Tukey’s post hoc test. ^(2)^ Kruskal–Wallis test with Conover’s post hoc test. Different uppercase letters indicate significant differences among resin composites within the same NSF concentration. Different lowercase letters indicate significant differences among the corresponding concentrations. * Statistically significant.

**Table 4:** Visual color evaluation.

Group	Control	1 wt%	2 wt%	4 wt%
**VA**	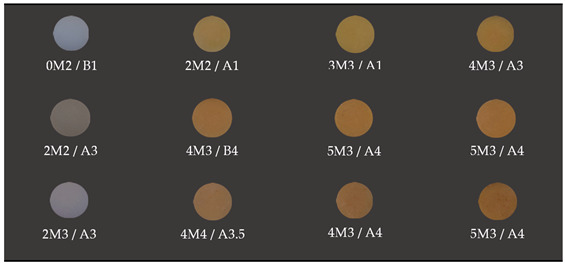			
**FB**				
**FS**				

VITA 3D-Master shade guide/VITA Classical A1–D4 shade.

## Data Availability

The data presented in this study are available on request from the corresponding author. The data are not publicly available due to privacy.

## Abbreviations

The following abbreviations are used in this manuscript:

AgNPs	silver nanoparticles
NSF	silver nanofluoride
FB	Filtek Bulk Fill Flowable
FS	Filtek Supreme Flowable
VA	Vitra APS Unique Flowable
Ag^+^	silver ions
ROS	reactive oxygen species
SeNPs	selenium nanoparticles
GONPs	graphene oxide nanoparticles
TiO_2_NPs	titanium dioxide nanoparticles
ZrO_2_NPs	zirconium dioxide nanoparticles
SiO_2_NPs	silicon dioxide nanoparticles
Ag@SiO_2_NPs	silica-coated silver nanoparticles
ppm	parts per million
wt%	weight percentage
APS	Advanced Polymerization System
BPA	bisphenol A
Bis-GMA	bisphenol A glycidyl methacrylate
Bis-EMA	ethoxylated bisphenol A dimethacrylate
Bis-DMA	bisphenol A dimethacrylate
UDMA	urethane dimethacrylate
TEGDMA	triethylene Glycol Dimethacrylate
PEGDMA	polyethylene glycol dimethacrylate
TEM	transmission electron microscopy
VHN	Vickers microhardness
CP	cross-polarization
UV-Vis	ultraviolet–visible
LSPR	localized surface plasmon resonance
FWHM	full width at half maximum
CIEDE2000	CIEDE2000 color difference formula
CIELAB	CIE Lab* color space
ΔE_00_	total color difference calculated using the CIEDE2000 formula
ΔE	total color difference calculated using the CIELAB system
PT	perceptibility threshold
AT	acceptability threshold
ANOVA	analysis of variance
SD	standard deviation
AgNO_3_	silver nitrate
NaBH_4_	sodium borohydride
NaF	sodium fluoride
